# Targeting PKM2 Enhances the Anti-Tumor Function of CD8^+^ T Cells Through Metabolic Reprogramming

**DOI:** 10.3390/ijms27177664

**Published:** 2026-08-27

**Authors:** Junxiu Zhang, Shuyi Wu, Qin Yin, Yanglin Liu, Shuguo Zheng, Peiwei Yang

**Affiliations:** 1 School of Pharmacy, Wannan Medical University, 22 Wenchang West Road, Higher Education Park, Wuhu 241002, China; zhangjunxiu@wnmc.edu.cn (J.Z.); 19965146187@163.com (S.W.); yq0553@163.com (Q.Y.); lylin20@163.com (Y.L.); 2 Pharmacology Laboratory of Traditional Chinese Medicine, Wannan Medical University, 22 Wenchang West Road, Higher Education Park, Wuhu 241002, China

**Keywords:** adoptive cell transfer, enolase 1, glycolysis, metabolic reprogramming, CD8^+^ T cells

## Abstract

The efficacy of adoptive cell transfer (ACT) therapy in solid tumors is often limited by the functional exhaustion and insufficient persistence of infused CD8^+^ T cells within the tumor microenvironment. Through the integrated analysis of single-cell transcriptomic data, this study identified enolase 1 (ENO1), a key rate-limiting enzyme in glycolysis, as a core gene highly correlated with the superior anti-tumor phenotype of tumor-infiltrating lymphocytes (TILs). However, in vitro functional validation demonstrated that the overexpression of *Eno1* failed to substantially enhance the anti-tumor efficacy of mouse T cells, suggesting the presence of a downstream metabolic regulatory node within the glycolytic cascade that restricts the conversion of carbon flux. To overcome this limitation, we introduced the small molecule activator TEPP-46 to target a crucial downstream metabolic hub, pyruvate kinase M2 (PKM2). Transcriptome sequencing confirmed that PKM2 activation successfully induced systemic metabolic rewiring in CD8^+^ T cells and broadly upregulated the expression of cytotoxicity- and memory-related genes. In an in vivo B16-OVA melanoma model, OT-1 T cells subjected to In vitro TEPP-46 pretreatment exhibited significantly enhanced tumor-suppressive capabilities and effectively promoted the preferential differentiation of T cells into central memory T cells (Tcm). In summary, this study highlights the importance of targeting downstream metabolic nodes to bypass intrinsic metabolic restrictions in T cells. It demonstrates that in vitro metabolic pretreatment via PKM2 activation represents an effective translational strategy for optimizing the anti-tumor efficacy of ACT cell products.

## 1. Introduction

Adoptive cell transfer (ACT), particularly tumor-infiltrating lymphocyte (TIL) therapy, has demonstrated tremendous potential in the clinical treatment of solid tumors such as melanoma [1,2]. However, its overall response rate across a broader range of solid tumors remains limited. A core obstacle is that the complex tumor microenvironment (TME) is characterized by severe metabolic competition and immunosuppressive networks. These conditions easily drive the infused CD8^+^ T cells into a state of exhaustion, manifested by impaired in vivo expansion, decreased secretion of effector molecules, and the upregulation of multiple inhibitory receptors [3,4,5]. Therefore, elucidating the key mechanisms that dictate the intrinsic anti-tumor fitness of CD8^+^ T cells, and thereby exploring intervention strategies to promote their long-term survival and memory differentiation in vivo, is urgently needed to overcome the current efficacy bottleneck of ACT.

The activation, proliferation, and anti-tumor effector functions of T cells are highly dependent on robust intracellular metabolic reprogramming [6]. In particular, glycolysis plays a fundamental role during the early activation and effector phases of T cells [7]. Glycolysis not only rapidly replenishes the ATP required by cells but also provides essential macromolecular precursors for massive clonal expansion and the synthesis of key effector molecules, such as IFN-γ and TNF-α [8,9]. Conversely, mitochondrial metabolic pathways, including oxidative phosphorylation (OXPHOS) and the tricarboxylic acid (TCA) cycle, are indispensable for maintaining global cellular energy homeostasis, promoting the development of Tcm, and supporting long-term survival [10]. Thus, distinct metabolic networks fulfill specific roles at different stages of the T cell life cycle, jointly sustaining the persistence of anti-tumor immune responses.

Enolase 1 (ENO1), a key rate-limiting enzyme in the glycolytic pathway, plays a central role in catalyzing the conversion of 2-phosphoglycerate to phosphoenolpyruvate [11]. Recent studies have revealed that metabolic enzymes participate not only in basic energy metabolism but also critically influence the fate determination and effector states of various immune cells through their expression levels and activity [12]. Although glycolysis is crucial for the early effector functions of CD8^+^ T cells and ENO1 serves as a critical node in maintaining this metabolic cascade, there is currently a lack of systematic investigation and in-depth mechanistic validation as to whether enhancing glycolytic flux by directly targeting upstream ENO1 can substantially improve the overall anti-tumor efficacy of CD8^+^ T cells in ACT.

In this study, through the integrated analysis of clinical scRNA-seq data, we identified the key glycolytic enzyme ENO1 as a core gene highly correlated with the superior anti-tumor efficacy of TILs. However, subsequent validation demonstrated that upregulation of ENO1 failed to substantially enhance the anti-tumor phenotype of T cells. This suggested that additional downstream metabolic constraints may limit the functional output of T cells. Accordingly, we utilized a small molecular activator to target a crucial downstream metabolic hub, pyruvate kinase M2 (PKM2). Our results show that the targeted activation of PKM2 successfully overcomes this bottleneck. By inducing systemic metabolic rewiring in CD8^+^ T cells, PKM2 activation effectively promoted their preferential differentiation into Tcm, ultimately leading to significantly enhanced in vivo tumor suppression in ACT. This study highlights the critical importance of targeting downstream metabolic nodes to bypass restrictions, thereby providing a novel, translatable strategy for the optimization of ACT cell products.

## 2. Results

### 2.1. ENO1 Is Highly Correlated with the Anti-Tumor Activity of CD8^+^ T Cells

**Figure 1:**
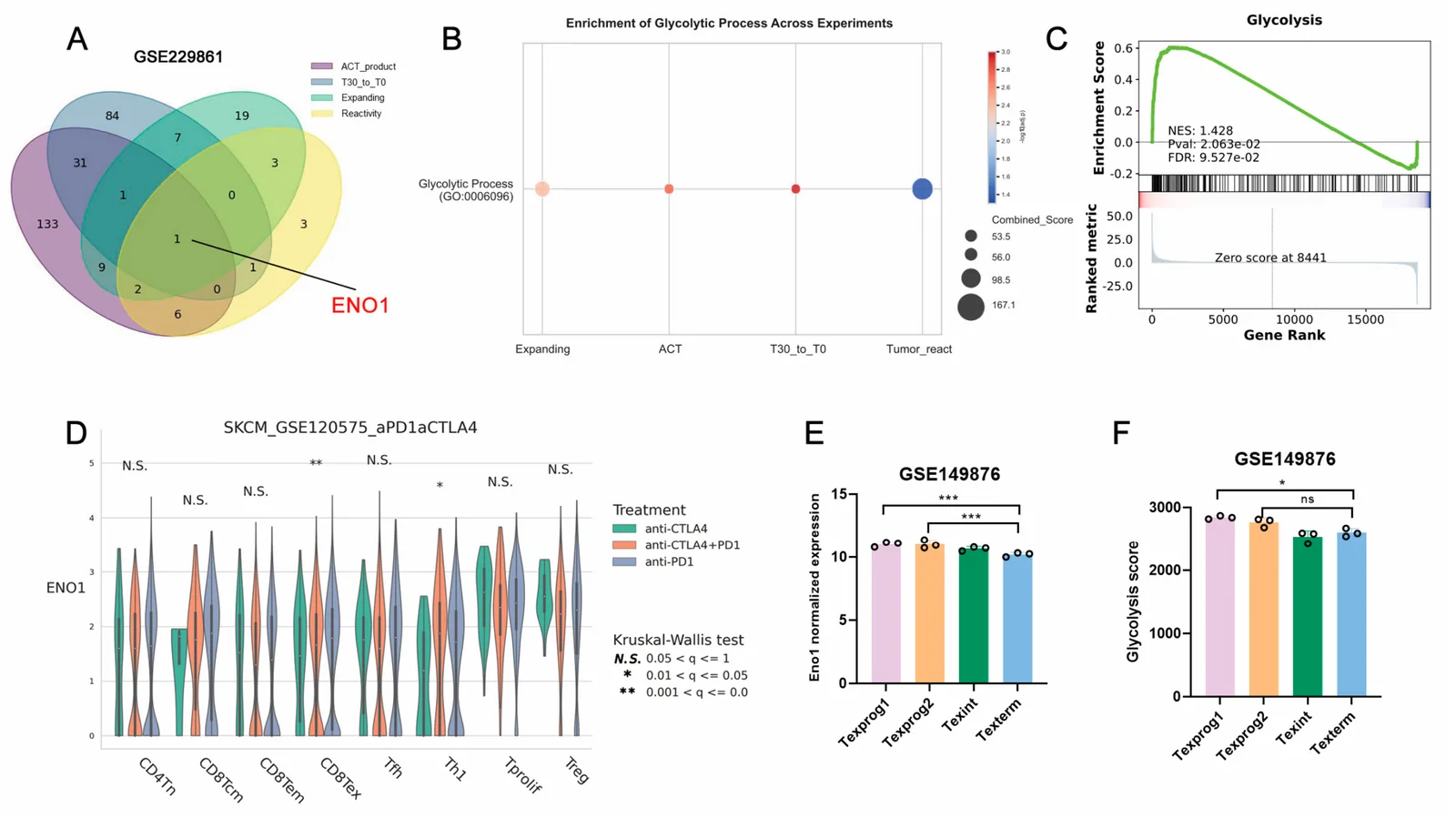
Identification of the rate-limiting glycolytic enzyme ENO1 as highly correlated with the anti-tumor phenotype of T cells. (**A**) Venn diagram showing the intersection of four highly expressed differentially expressed gene (DEG) sets (ACT_product, T30_to_T0, Reactivity, Expanding), identifying the core gene *ENO1*. (**B**) Gene Ontology (GO) enrichment analysis of the aforementioned highly expressed DEG sets. (**C**) Gene Set Enrichment Analysis (GSEA) of the glycolysis pathway based on transcriptomic data from immunotherapy-responding patients (T30_to_T0). (**D**) Comparison of *ENO1* expression abundance in T cell subpopulations of patients receiving immune checkpoint therapy, derived from public single-cell databases. (**E**,**F**) Comparison of *Eno1* expression levels and overall glycolysis signal intensity between murine CD8^+^ progenitor exhausted T cells and terminally exhausted T cells. ns, *p* > 0.05, *, *p* < 0.05, ***, *p* < 0.001.

To identify key genes influencing the anti-tumor efficacy of TIL therapy, we conducted an integrated analysis of clinical scRNA-seq data derived from a systematic study of TIL therapy. We extracted four distinct differentially expressed gene (DEG) sets associated with superior anti-tumor functions: genes highly expressed in ACT products that yielded better post-infusion outcomes (ACT_product), genes upregulated at day 30 post-treatment compared to baseline in responding patients (T30_to_T0), genes exhibiting high tumor reactivity in vitro (Reactivity), and genes associated with high in vitro expansion capacity (Expanding). A Venn diagram intersection revealed that *ENO1* was the only core gene significantly highly expressed across all four gene sets (Figure 1A). Given that ENO1 is a key rate-limiting enzyme in the glycolysis pathway, we performed Gene Ontology (GO) enrichment analysis on these gene sets, confirming that the glycolysis pathway was significantly enriched (Figure 1B). Furthermore, Gene Set Enrichment Analysis (GSEA) on the complete sequencing dataset also demonstrated a significant enrichment of glycolysis-related genes in the T30_to_T0 group (Figure 1C). To further investigate the expression pattern of *ENO1* in the context of immunotherapy, we queried public scRNA-seq databases. The analysis showed that in patients receiving anti-PD-1 therapy, *ENO1* expression was significantly upregulated in CD8^+^ exhausted T cells (Tex) and Th1 cells compared to other treatment groups. This suggests that the activation of this gene may be closely associated with the clinical benefits of immunotherapy (Figure 1D). In addition, considering that distinct CD8^+^ T cell subsets play varying roles in anti-tumor immunity, we re-analyzed a sequencing dataset of murine CD8^+^ T cell subsets. The results indicated that, compared to terminally exhausted T cells (Tex term), progenitor exhausted T cells (Texprog) not only exhibited higher *Eno1* expression but also displayed a stronger overall glycolytic signature (Figure 1E,F). Collectively, these findings suggest that ENO1 and its mediated glycolytic metabolism likely play a crucial role in maintaining the anti-tumor activity of CD8^+^ T cells.

### 2.2. Effects of Eno1 Overexpression on Glycolysis and In Vitro Anti-Tumor Functions of CD8^+^ T Cells

**Figure 2:**
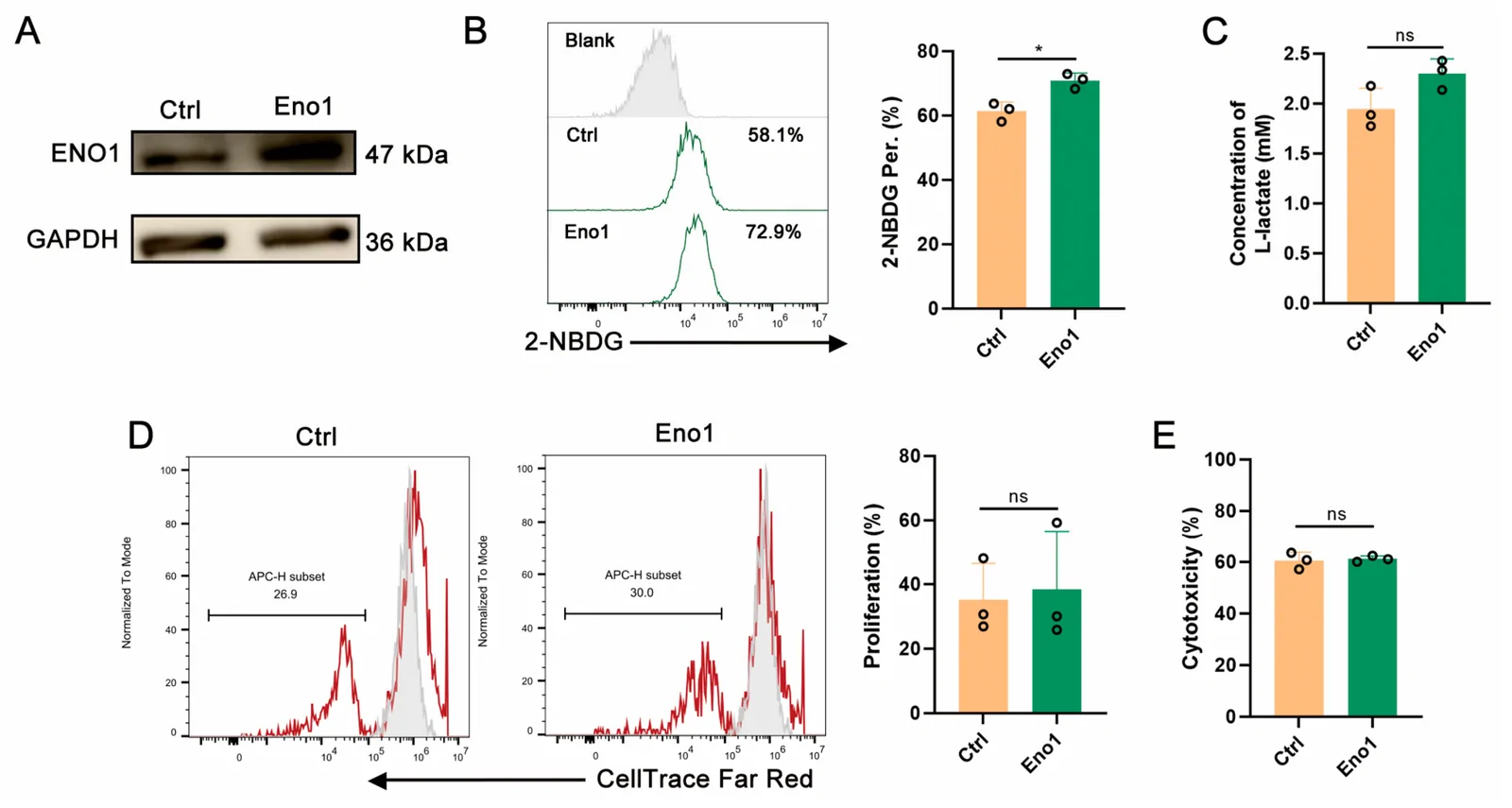
Overexpression of *Eno1* has limited effects on the glycolytic flux and in vitro anti-tumor functions of CD8^+^ T cells. (**A**) Western blot analysis of ENO1 protein expression levels in OT-1 T cells of the control group (Ctrl) and the *Eno1* overexpression group (Eno1). (**B**) Flow cytometric evaluation of the uptake capacity for the 2-NBDG in both groups of T cells (Grey histograms represent the blank controls). (**C**) Quantitative detection of L-lactate secretion in the culture supernatants of both groups of T cells after activation. (**D**) in vitro expansion capacity of both groups of T cells co-incubated with B16-OVA target cells, evaluated using the CellTrace assay. (**E**) Specific killing efficiency of both groups of T cells against B16-OVA target cells, measured via a luminescence-based substrate assay. ns, *p* > 0.05, *, *p* < 0.05.

To directly evaluate the impact of *Eno1* on T cell function in vitro, we isolated splenic CD8^+^ T cells from OT-1 mice and constructed *Eno1* overexpressing cells (Eno1) using retroviral transduction, with empty vector transfected T cells serving as controls (Ctrl). Western blot results confirmed a significant increase in ENO1 protein expression in the OT-1 T cells of the overexpression group (Figure 2A). Subsequently, under CD3/CD28 antibody stimulation, we assessed cellular glucose uptake using the fluorescent glucose analog 2-NBDG. Flow cytometry revealed that *Eno1* overexpression significantly enhanced the uptake of 2-NBDG by T cells (Figure 2B). However, no statistical difference was observed between the two groups regarding L-lactate secretion levels in the culture supernatant (Figure 2C). For functional evaluation, we co-incubated both groups of OT-1 T cells with B16-OVA cells. CellTrace proliferation assays indicated that *Eno1* overexpression did not significantly alter the in vitro expansion capacity of T cells (Figure 2D). Concurrently, luminescence-based in vitro cytotoxicity assays also showed no significant difference in the killing efficiency against target cells between the two groups (Figure 2E). These data suggest that although the upregulation of *Eno1* can partially promote glucose metabolic flux, it is insufficient to translate into a substantial enhancement of the in vitro tumor cell-killing capacity.

### 2.3. Effects of Eno1 Knockdown on Glycolysis and In Vitro Anti-Tumor Functions of CD8^+^ T Cells

**Figure 3:**
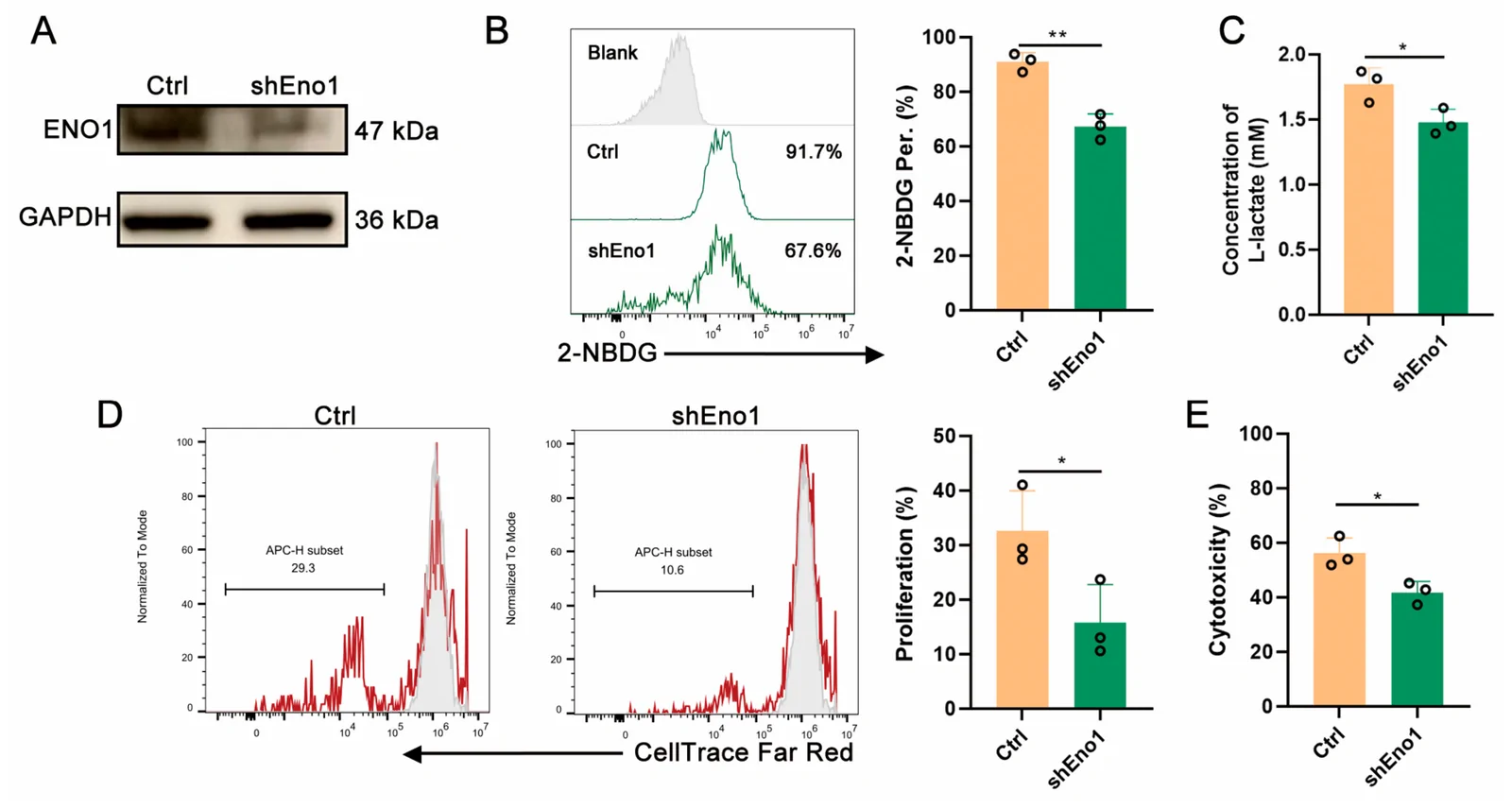
Knockdown of *Eno1* significantly impairs the glycolytic capacity and anti-tumor activity of CD8^+^ T cells. (**A**) Western blot analysis confirming the silencing of ENO1 protein in OT-1 T cells following shRNA-targeted knockdown (shEno1). (**B**) Flow cytometric analysis of 2-NBDG uptake capacity in T cells after *Eno1* knockdown (Grey histograms represent the blank controls). (**C**) Quantitative analysis of the difference in L-lactate concentration in the culture supernatants between the *Eno1* knockdown and control groups. (**D**) Evaluation of the impact of *Eno1* deficiency on antigen-specific T cell proliferation using the CellTrace assay. (**E**) Determination of the effect of *Eno1* deficiency on the in vitro target cell killing efficacy of T cells using a luminescence-based assay. *, *p* < 0.05, **, *p* < 0.01.

To clarify the necessity of endogenous *Eno1* in maintaining CD8^+^ T cell function, we specifically knocked down the *Eno1* gene in OT-1 T cells using shRNA and confirmed target protein silencing via Western blot (Figure 3A). Under antibody activated conditions, *Eno1* knockdown (shEno1) resulted in a significant decrease in 2-NBDG uptake by T cells compared to the control group (Figure 3B), which was accompanied by a marked reduction in L-lactate concentration in the cell supernatant (Figure 3C). These results indicate that ENO1 is an important regulator required for maintaining optimal glycolytic activity in T cells. At the functional level, co-incubation assays with B16-OVA target cells further revealed that *Eno1* deficiency not only inhibited antigen-specific T cell proliferation (Figure 3D) but also led to a significant decrease in their in vitro tumor-killing efficacy (Figure 3E). Together, these forward and reverse experiments demonstrate that ENO1 is a critical node mediating the glycolytic cascade in CD8^+^ T cells, and its functional loss severely impairs their in vitro tumor cell-killing capacity.

### 2.4. Activation of the Downstream Kinase PKM2 Induces Metabolic Rewiring and Enhances Anti-Tumor Functions in T Cells

**Figure 4:**
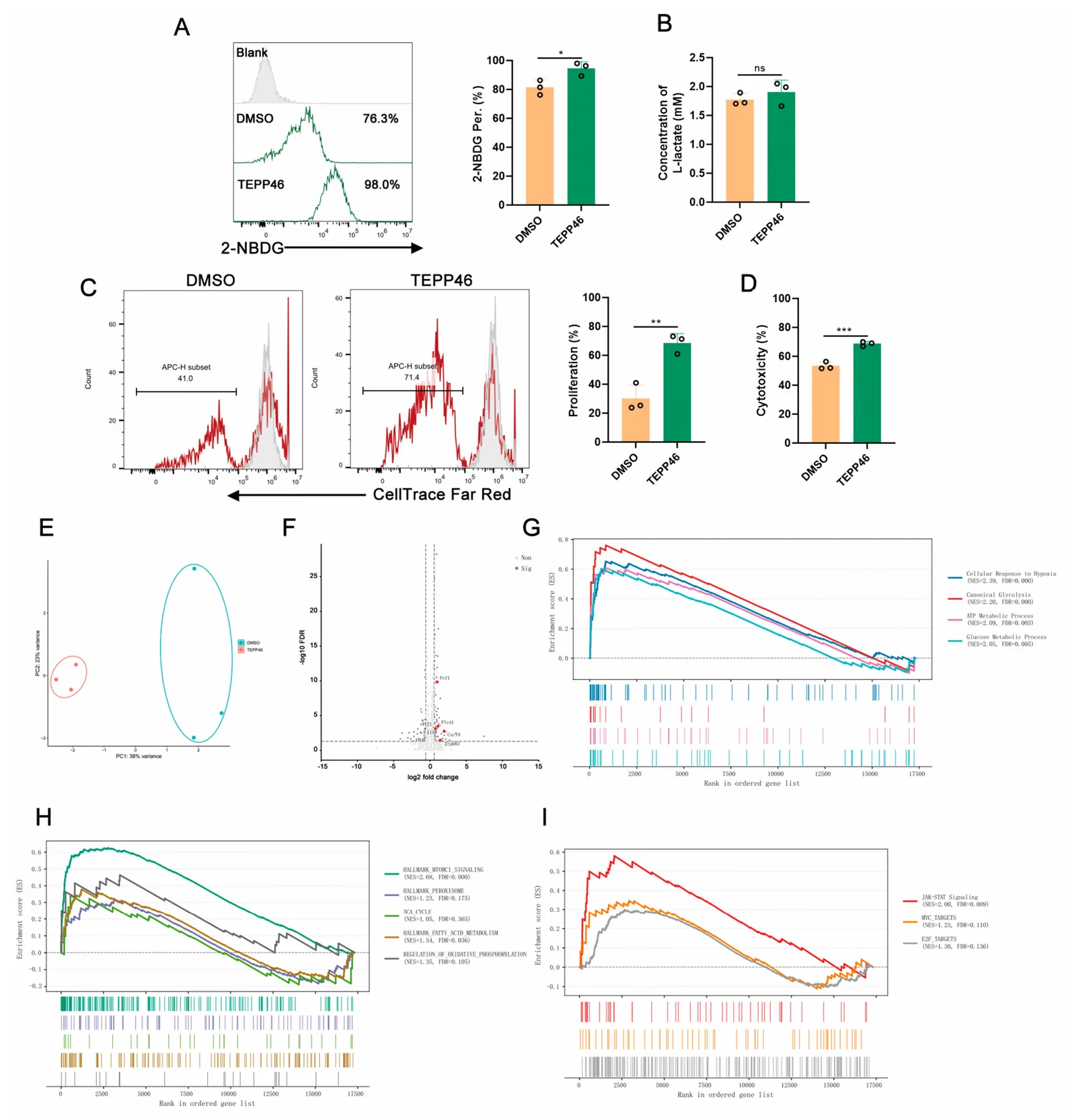
TEPP46 induces global metabolic rewiring and enhances the anti-tumor functions of T cells. Quantitative detection of (**A**) 2-NBDG uptake capacity (Grey histograms represent the blank controls) and (**B**) L-lactate secretion levels in the culture supernatants of CD8^+^ T cells treated with TEPP46 or vehicle. (**C**) Specific proliferative vitality and (**D**) target cell killing efficacy of the TEPP46-treated and control groups after co-incubation with B16-OVA cells. (**E**) Principal component analysis (PCA) of RNA-seq transcriptomic data from both groups of T cells following co-incubation. (**F**) DEGs analysis demonstrating the upregulation of core genes related to cytotoxicity, memory differentiation, and tissue residency induced by TEPP46 treatment. (**G**–**I**) GSEA results revealing that TEPP46 treatment significantly enriches pathways associated with energy metabolism and effector functions. ns, *p* > 0.05, *, *p* < 0.05, **, *p* < 0.01, ***, *p* < 0.001.

Based on our finding that *Eno1* overexpression offers limited improvement to CD8^+^ T cell function, we hypothesized that ENO1 might not be the primary metabolic bottleneck restricting function within the T cell glycolytic cascade. Therefore, we shifted our intervention target downstream to a key enzyme PKM2, and utilized the small molecular activator TEPP46 to specifically enhance PKM2 activity [13]. Metabolic assays showed that TEPP46 treatment significantly increased 2-NBDG uptake in CD8^+^ T cells (Figure 4A) but did not alter lactate secretion levels (Figure 4B). More importantly, functional assays demonstrated that T cells pretreated with TEPP46 exhibited significantly enhanced proliferative vitality and target cell killing capacity when co-incubated with B16-OVA cells (Figure 4C,D). To further elucidate the molecular mechanisms underlying the TEPP46-mediated efficacy enhancement, we performed RNA-seq analysis on the co-incubated T cells. Principal Component Analysis (PCA) showed a distinct separation at the transcriptomic level between the drug-treated and control groups (Figure 4E). Analysis of DEGs revealed that the TEPP46 treatment group significantly upregulated genes associated with cytotoxicity (*Prf1*), memory-related cytokines (*Il10*, *Il21*), T cell chemokines (*Cxcl9*), as well as T cell activation (*Plcd1*) and tissue residency (*Zfp683*). This indicates a differentiation shift toward a superior anti-tumor phenotype (Figure 4F). GSEA further revealed that TEPP-46 not only upregulated pathways related to glycolysis, glucose metabolism, and ATP synthesis but also significantly enriched pathways involving oxidative phosphorylation (OXPHOS), the tricarboxylic acid (TCA) cycle, fatty acid metabolism, and peroxisomes (Figure 4G,H). This systemic metabolic reprogramming may also explain the absence of increased lactate production, suggesting that activation of PKM2 by TEPP46 is associated with altered glucose metabolism and metabolic programs. This active metabolic state concurrently fortified the anti-tumor functions of CD8^+^ T cells, manifested by the activation of JAK-STAT, MYC-targeted, and E2F-targeted signaling pathways, all of which are closely correlated with anti-tumor efficacy (Figure 4I).

### 2.5. TEPP46 Pretreatment Enhances In Vivo Tumor-Suppressive Efficacy

**Figure 5:**
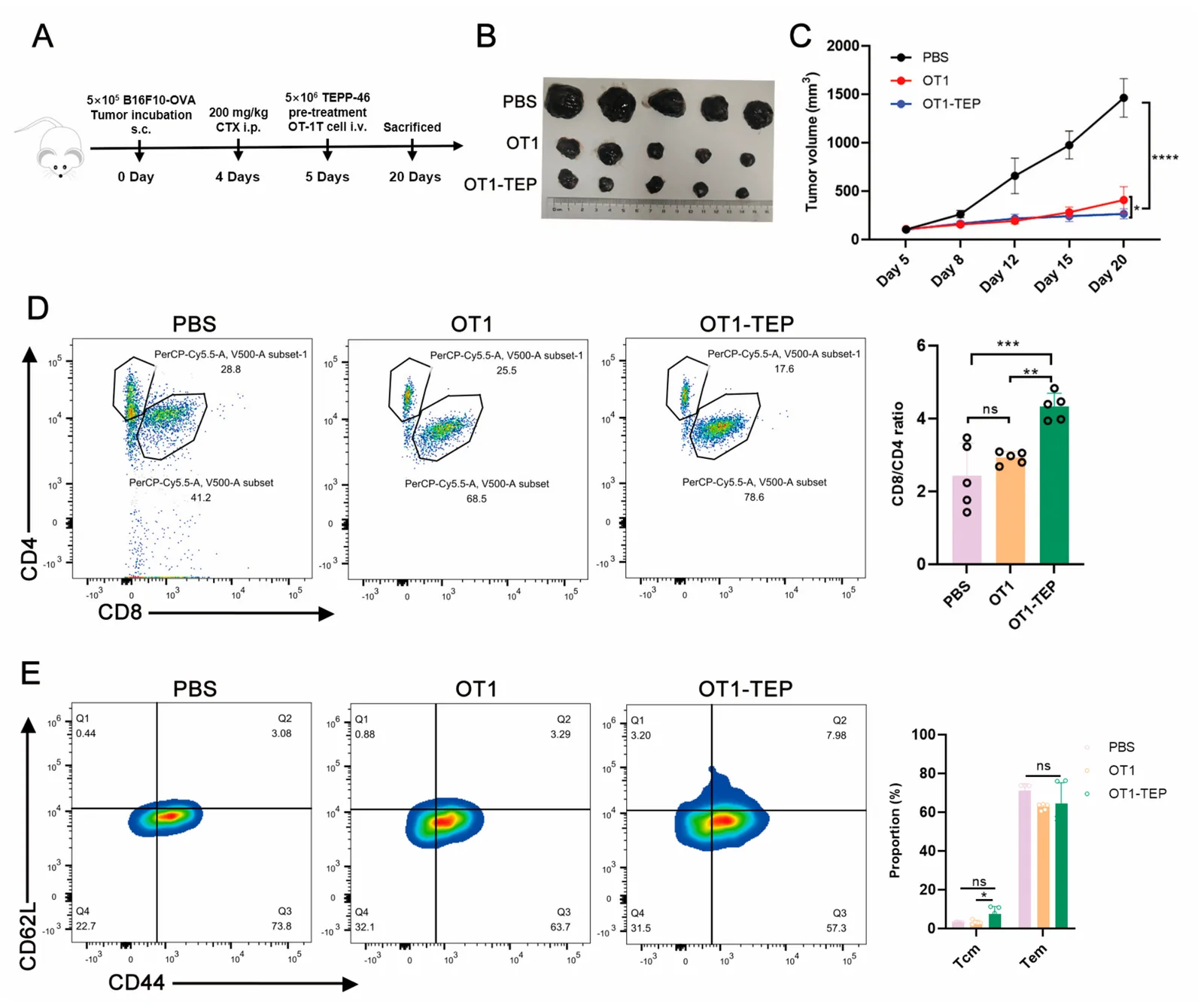
In vitro TEPP46 pretreatment promotes the differentiation of infused T cells into a central memory phenotype and enhances in vivo tumor-suppressive efficacy. (**A**) Schematic workflow of mouse in vivo experiments (n = 5, two independent experiments were performed). (**B**) Photographs of tumors from each group of mice at the experimental endpoint. (**C**) In vivo tumor growth curves of tumor-bearing mice following the infusion of OT-1 T cells from either the control or TEPP46 pretreatment group. (**D**) Flow cytometric analysis of the ratio of CD8^+^ to CD4^+^ T cells among tumor-infiltrating lymphocytes in each group of mice. (**E**) Flow cytometric evaluation of proportional changes in memory differentiation subpopulations (CD62L^+^CD44^+^ central memory Tcm, CD62L^−^CD44^+^ effector memory Tem) among tumor-infiltrating CD8^+^ T cells. ns, *p* > 0.05, *, *p* < 0.05, **, *p* < 0.01, ***, *p* < 0.001, ****, *p* < 0.0001.

To validate the clinical translational potential of in vitro metabolic interventions for ACT therapy, we evaluated the impact of TEPP46 pretreatment on the in vivo tumor-suppressive function of OT-1 T cells in a tumor bearing preclinical model (Figure 5A). Tumor growth curves and endpoint dissection results showed that, compared to the infusion of control T cells, OT-1 T cells pretreated with TEPP46 exhibited a stronger in vivo tumor inhibitory capacity (Figure 5B,C).Flow cytometric analysis of TIL revealed a significantly higher ratio of infiltrating CD8^+^ to CD4^+^ T cells in the TEPP46 pretreatment group, indicating a shift toward a CD8^+^ T-cell–dominant intratumoral immune profile (Figure 5D). Additionally, we examined the memory differentiation phenotypes of tumor-infiltrating CD8^+^ T cells. We found that the proportion of Tcm in the TEPP46 pretreatment group was significantly increased compared to both the control and untreated groups, whereas the proportions of effector memory (Tem) showed no obvious changes (Figure 5E). These findings indicate that metabolic rewiring induced by PKM2 activation can effectively promote the differentiation of infused T cells into Tcm cells, which may contribute to sustained tumor control, whereas transient differences in Tem responses at earlier stages cannot be excluded because longitudinal phenotyping was not performed.

## 3. Discussion

The major challenge of ACT therapy in solid tumors is the difficulty for infused CD8^+^ T cells to maintain long-term survival and an anti-tumor phenotype in the hostile TME [14]. Starting with unbiased data mining of a large, systematic clinical cohort, this study identified the rate-limiting glycolytic enzyme ENO1 as an important target highly correlated with superior TIL efficacy. However, in vitro validation indicated that the sole upregulation of ENO1 expression was insufficient to substantially enhance the anti-tumor activity of T cells. Based on this finding, we further targeted the key downstream metabolic hub—PKM2. By using the small molecular activator TEPP-46, we successfully broke the downstream metabolic regulatory node. Inducing deep metabolic rewiring in CD8^+^ T cells significantly promoted their preferential differentiation into Tcm, thereby improving their anti-tumor efficacy in both in vitro and in vivo models. This study provides strong theoretical support and experimental evidence for optimizing ACT cell products through precise metabolic interventions.

The transition of intervention targets from ENO1 to PKM2 in this study profoundly reveals the phenomenon of a downstream metabolic regulatory node within the T cell glycolytic cascade. During the early stage of T cell activation, glycolysis is crucial for the rapid generation of macromolecular precursors and effector molecules [8,15]. However, simply driving carbon flux by upregulating upstream rate-limiting enzymes (like ENO1) often leads to a blind accumulation of intracellular intermediate metabolites or their accelerated conversion into lactate. Such intense and isolated aerobic glycolysis (the Warburg effect) has been proven to be a major factor driving T cells toward terminal exhaustion [16,17,18]. In contrast, PKM2 is situated at the branch point between glycolysis and mitochondrial metabolism [19]. TEPP-46 can induce the transition of PKM2 from a low-activity dimer to a high-activity tetramer, accelerating the conversion of phosphoenolpyruvate (PEP) to pyruvate, and effectively channeling it into the mitochondria for OXPHOS and the TCA cycle [20]. This systemic diversion not only avoids the excessive accumulation of lactate but also reshapes the energy homeostasis of T cells. This explains why targeting a downstream metabolic hub can achieve superior phenotypic transformations compared to upstream interventions.

RNA-seq and GSEA further corroborated the global changes in T cell phenotype brought about by this metabolic rewiring. The TEPP-46-treated group exhibited not only significant upregulation of the OXPHOS and TCA cycle pathways but also widespread activation of cytotoxicity (Prf1), memory and tissue-residency traits (Zfp683, Il10, Il21), and the JAK-STAT signaling pathway. Although this study primarily characterized the phenotypic advantages induced by metabolic rewiring at the transcriptional level, the underlying molecular coupling mechanisms remain to be further elucidated. Moreover, because PKM2 was primarily investigated through pharmacological activation with TEPP-46, further genetic validation using PKM2 loss-of-function approaches would strengthen the causal link between PKM2 activity and the observed T-cell phenotypes. Recent studies have shown that the “metabolite-epigenetic” regulatory axis plays a core role in maintaining the T cell memory network [21]. Active mitochondrial metabolism increases the production of key metabolic intermediates (such as acetyl-CoA and α-ketoglutarate) [22,23]. These small molecules are vital substrates for histone acetyltransferases and DNA demethylases, respectively, which directly activate the transcriptional programs of memory-related genes (like Tcf7) through chromatin remodeling [24]. In the future, combining isotope tracing analysis with single-cell multi-omics (such as scATAC-seq [25]) will help systematically dissect the precise epigenetic mechanisms by which PKM2 activation rewires the T cell memory network.

Regarding clinical translation strategies, this study opted for an in vitro “pre-treatment” approach rather than in vivo combination therapy. This choice was not only based on biological mechanisms but also holds considerable translational potential. First, several cutting-edge studies have indicated that the optimal intervention window for targeting PKM2 to rewire CD8^+^ T cell mitochondrial metabolism is during the early stages of antigen-stimulated T cell activation. The concurrent addition of TEPP-46 during the in vitro expansion phase perfectly aligns with this early metabolic reprogramming window [13]. Second, because TEPP-46 may affect the metabolic state of tumor cells or myeloid-derived suppressor cells in the TME [26], in vitro pre-treatment eliminates the potential systemic off-target effects associated with in vivo administration, confirming that the tumor-suppressive advantage stems solely from the intrinsic metabolic optimization of CD8^+^ T cells. However, the tumor-suppressive advantage of pre-treated T cells did not increase significantly further in the in vivo models. This may be because the infused T cells, once deprived of in vitro drug maintenance, are rapidly exposed to the hypoxic, nutrient-deprived, and immunosuppressive microenvironment of solid tumors, causing their established metabolic advantage to gradually diminish. To address this limitation, future optimizations of ACT could take two paths: first, administering a short-term, low-dose metabolic modulating drug during the early post-infusion period to consolidate the rewiring effects. Second, combining the metabolically optimized T cell therapy with immune checkpoint blockade (such as anti-PD-1 monoclonal antibodies). PD-1 blockade can effectively alleviate the overall metabolic competition in the TME and relieve inhibitory signals. This provides a more favorable killing environment for the highly metabolically adaptable pre-treated T cells, achieving a dual-strike efficacy through both intrinsic T cell metabolic rewiring and extrinsic microenvironmental improvement. Nevertheless, as the present in vivo findings were obtained using a B16-OVA tumor model and murine T cells, further validation in additional tumor models and human T-cell systems will be necessary to establish the generalizability and translational relevance of this strategy.

In summary, this study not only elucidates the scientific necessity of overcoming the upstream “metabolic bottleneck” in T cells but also demonstrates that achieving systemic metabolic rewiring by activating the key downstream hub, PKM2, can effectively promote the differentiation of CD8^+^ T cells into central memory phenotypes. This in vitro pre-treatment strategy, based on metabolic pathway optimization, exhibits tremendous potential for direct integration into existing clinical TIL or CAR-T manufacturing protocols, offering an innovative translational perspective for developing the next generation of ACT with long-lasting tumor-suppressive activity.

## 4. Materials and Methods

### 4.1. Animals and Ethics Statement

Wild-type C57BL/6 mice and OT-1 mice containing a transgenic T cell receptor (TCR) specifically recognizing the OVA antigen (female, 5–6 weeks old) were purchased from Cyagen Biosciences (Suzhou, China). All mice were housed in a specific pathogen-free (SPF) standard animal facility with free access to food and water. All animal experimental procedures were executed strictly in accordance with the guidelines and approved protocols of the Animal Care and Use Committee of Wannan Medical University.

### 4.2. Cell Lines and Culture

The HEK-293T cell line and the mouse melanoma cell line stably expressing luciferase and the OVA antigen (B16-OVA-luciferase) were cultured in DMEM or RPMI-1640 medium supplemented with 10% fetal bovine serum (Gibco, Grand Island, NY, USA), 100 U/mL penicillin, and 100 μg/mL streptomycin. Cells were routinely subcultured in a humidified incubator at 37 °C with 5% CO_2_.

### 4.3. Bioinformatics Analysis

DEGs analysis was performed on a subset of sequencing data from the GSE229861 [27] dataset to extract a set of significantly upregulated genes, and a Venn diagram was generated using the matplotlib-venn module in Python (3.10) to identify the core overlapping genes. Gene Ontology (GO) functional enrichment analysis of the intersected genes was conducted using the ClusterProfiler package in R (4.2.3) [28]. Furthermore, GSEA was performed using the GSEApy (v1.3.0) [29] to evaluate the enrichment level of the glycolysis pathway. The normalized expression profiles of *ENO1* across different cell subpopulations in immunotherapy cohorts were retrieved from the Tumor Immune Single-cell Hub 2 (TISCH2) [30] database. For the GSE149876 dataset [31], the normalized expression values of *Eno1* in specific T cell subtypes were extracted, and the glycolysis score for each cell cluster was calculated using the single-sample GSEA (ssGSEA) algorithm.

### 4.4. Plasmid Construction and Retroviral Packaging

The recombinant vectors for *Eno1* overexpression and the shRNA vectors for *Eno1* knockdown were constructed and sequence-verified by General Biol (Anhui, China). Retroviral packaging was performed in HEK-293T cells: the MSCV viral backbone plasmid and the pCL-Eco packaging plasmid were co-transfected into HEK-293T cells at a mass ratio of 3:1 using the EZTrans transfection reagent (Life-iLab, Shanghai, China). At 72 h post-transfection, the culture supernatant containing the retrovirus was collected, filtered through a 0.45 μm membrane, and used for subsequent T cell infection or aliquoted and stored at −80 °C.

### 4.5. T Cell Isolation, Activation, and Retroviral Transduction

Spleens from OT-1 mice were aseptically isolated, and mouse splenic lymphocytes were obtained using a Mouse Lymphocyte Isolation Kit (Solarbio, Beijing, China). Primary mouse T cells were sorted and purified using a MACS magnetic separation column with a fluorescent anti-CD8-APC antibody (BioLegend, San Diego, CA, USA) and Anti-APC MicroBeads (Miltenyi Biotec, Bergisch Gladbach, Germany) according to the manufacturers’ instructions. The sorted T cells were seeded into 24-well plates pre-coated with anti-CD3 (0.5 μg/mL) and anti-CD28 (5 μg/mL) antibodies (BioLegend) and activated for 48 h. Subsequently, retroviral supernatant containing an appropriate concentration of Polybrene (8 μg/mL; Sigma-Aldrich, St. Louis, MO, USA) was added, and spinfection was performed by centrifugation at 1200× *g* for 1 h at 32 °C. Post-infection, the cells were cultured in RPMI-1640 complete medium supplemented with 200 U/mL IL-2.

### 4.6. Western Blotting

On day 5 post-infection, T cells were harvested, and total proteins were extracted using RIPA lysis buffer containing PMSF and a protease inhibitor cocktail (Beyotime, Shanghai, China). After determining the protein concentration using a BCA Protein Assay Kit (Beyotime), equal amounts of protein samples were separated by SDS-PAGE and wet-transferred onto PVDF membranes. Following blocking with 5% non-fat milk for 1 h, the membranes were incubated overnight at 4 °C with a primary anti-ENO1 antibody (1:1000 dilution; Proteintech, Rosemont, IL, USA) or anti-GAPDH antibody (1:2000 dilution; Proteintech). The following day, the membranes were washed with TBST and incubated with a corresponding HRP-conjugated secondary antibody (1:20,000 dilution; Proteintech) for 1 h. Finally, chemiluminescence detection was performed using ECL substrate on a Cytiva gel imaging system (Marlborough, MA, USA).

### 4.7. Glucose Uptake Assay

T cells on day 5 post-infection were collected and transferred to antibody-coated plates for 24 h of stimulation. Subsequently, the cells were resuspended in a glucose-free starvation solution (PBS supplemented with 10% FBS) containing 100 μM 2-NBDG (Invitrogen, Carlsbad, CA, USA) and incubated at 37 °C in the dark for 30 min. After incubation, the cells were washed with cold PBS, and the intracellular 2-NBDG fluorescence intensity was immediately measured via flow cytometry to evaluate glucose uptake capacity.

### 4.8. Lactate Assay

T cells on day 5 post-infection were collected and stimulated in antibody-coated plates for 24 h, after which the cell culture supernatant was harvested. The concentration of L-lactate in the supernatant was measured using a WST-8 Lactate Assay Kit (Beyotime) and read on the Infinite M200 PRO microplate reader (Tecan, Männedorf, Switzerland) according to the manufacturer’s instructions.

### 4.9. In Vitro Proliferation and Cytotoxicity Assays

OT-1 T cells were collected on day 5 post-infection and labeled with CellTrace proliferation dye (Invitrogen, Carlsbad, CA, USA). The labeled effector T cells were co-incubated with target B16-OVA-luciferase cells at an effector-to-target (E:T) ratio of 2:1 for 24 h. After co-incubation, the suspension cells were collected, and the fluorescence dilution of CellTrace was analyzed by flow cytometry to evaluate the in vitro expansion capacity of the T cells.

For the cytotoxicity analysis, after 24 h of co-culture, the ONE-Glo Luciferase Assay System reagent (Vazyme, Nanjing, China) was added to the wells, and the luminescence values were recorded using the Infinite M200 PRO reader (Tecan, Männedorf, Switzerland). The target cell-specific cytotoxicity was calculated using the following formula:

$$\mathrm{Cytotoxicity}(\%)=\left(1-\frac{{\mathrm{Luminescence}}_{\mathrm{experimental}}}{{\mathrm{Luminescence}}_{\mathrm{target}\;\mathrm{only}}}\right)\times 100$$

### 4.10. RNA Sequencing and Transcriptomic Analysis

Sorted and purified primary T cells were activated in anti-CD3/CD28 coated plates for 48 h in the presence of 50 μM TEPP-46 (pre-treatment group) or an equal volume of DMSO (control group). They were then co-incubated with B16-OVA cells at an E:T ratio of 2:1 for 24 h. Following co-culture, T cells were re-purified using magnetic beads, and total RNA was extracted using TRIzol. The RNA samples were submitted to Genedenovo Biotechnology (Guangzhou, China) for library construction and transcriptome sequencing. The raw Fastq sequencing data were quantified for transcript expression using Salmon v1.12.0 software and mapped to the mouse reference genome (GRCm39). Differential expression analysis was performed using the R package DESeq2 (with thresholds set at |log2FC| > 0.585 and padj < 0.05). Pathway enrichment analysis and visualization were conducted using the GSEApy library in a Python environment.

### 4.11. In Vivo Tumor Models and Adoptive Cell Transfer

C57BL/6 mice were subcutaneously inoculated with 5 × 10^5^ B16-OVA tumor cells on the right flank. When the tumor volume reached approximately 80 mm^3^, the mice received an intraperitoneal injection of 200 mg/kg cyclophosphamide for pre-conditioning lymphodepletion. Twenty-four hours later, 5 × 10^6^ OT-1 T cells from either the control group or the TEPP-46 pre-treatment group were intravenously infused into each mouse via the tail vein. Tumor growth was continuously monitored using digital calipers. Once the experiment reached the ethical endpoint, the mice were euthanized, and the tumor tissues were harvested.

### 4.12. Flow Cytometry

Tumor tissues were minced and digested in a dissociation solution containing Collagenase I (0.5 mg/mL), Collagenase IV (0.5 mg/mL), and DNase I (1 mg/mL) (Solarbio, Beijing, China) at 37 °C to prepare single-cell suspensions. The cells were stained with a Live/Dead viability dye and fluorochrome-conjugated antibodies, including anti-CD45-FITC, CD3-PE-Cy7, CD4-BV510, CD8-PerCP5.5, CD44-APC, and CD62L-PE (BioLegend). Detection were performed using CytoFlex (Beckman Coulter, Brea, CA, USA). All flow cytometry data were analyzed using FlowJo software (v 10.6.2) (Tree Star, Ashland, OR, USA).

### 4.13. Statistical Analysis

Experimental data are presented as Mean ± SD. All statistical analyses were performed using GraphPad Prism software (Version 9) (San Diego, CA, USA). Comparisons between two groups were analyzed using the unpaired Student’s *t*-test. Univariate comparisons among three or more groups were performed using one-way analysis of variance (ANOVA). Bivariate data involving time and grouping, such as tumor growth curves, were analyzed using two-way ANOVA. A *p*-value < 0.05 was considered to indicate a statistically significant difference.

## Data Availability

The raw data supporting the conclusions of this article will be made available by the authors on request.

## Abbreviations

The following abbreviations are used in this manuscript:

ACT	Adoptive cell transfer
TIL	Tumor-infiltrating lymphocyte
TME	Tumor microenvironment
IFN-γ	Interferon gamma
OXPHOS	Oxidative phosphorylation
TCA	Tricarboxylic acid
Tcm	Central memory T cells
ENO1	Enolase 1
scRNA-seq	Single-cell RNA sequencing
PKM2	Pyruvate kinase M2
DEGs	Differentially expressed genes
GO	Gene Ontology
GSEA	Gene Set Enrichment Analysis
PD-1	Programmed cell death protein 1
Tex	Exhausted T cells
Tpex	Progenitor exhausted T cells
shRNA	Short hairpin RNA
PCA	Principal Component Analysis
Tem	Effector memory T cells
PEP	Phosphoenolpyruvate
CAR T	Chimeric antigen receptor T cell
FBS	Fetal bovine serum
TISCH2	Tumor Immune Single-cell Hub 2
ssGSEA	Single-sample Gene Set Enrichment Analysis
RIPA	Radioimmunoprecipitation assay
BCA	Bicinchoninic acid
SDS-PAGE	Sodium dodecyl sulfate-polyacrylamide gel electrophoresis
HRP	Horseradish peroxidase
